# Climate and seasonality drive the richness and composition of tropical fungal endophytes at a landscape scale

**DOI:** 10.1038/s42003-021-01826-7

**Published:** 2021-03-09

**Authors:** Shuzo Oita, Alicia Ibáñez, François Lutzoni, Jolanta Miadlikowska, József Geml, Louise A. Lewis, Erik F. Y. Hom, Ignazio Carbone, Jana M. U’Ren, A. Elizabeth Arnold

**Affiliations:** 1 School of Plant Sciences, University of Arizona, Tucson, AZ USA; 2 Independent Researcher, Gamboa, Republic of Panama; 3 Department of Biology, Duke University, Durham, NC USA; 4 MTA-EKE Lendület Environmental Microbiome Research Group, Eszterházy Károly University, Eger, Hungary; 5 Department of Ecology and Evolutionary Biology, University of Connecticut, Storrs, CT USA; 6 Department of Biology, Center for Biodiversity and Conservation Research, University of Mississippi, University, MS USA; 7 Center for Integrated Fungal Research, Department of Entomology and Plant Pathology, North Carolina State University, Raleigh, NC USA; 8 Department of Biosystems Engineering and BIO5 Institute, University of Arizona, Tucson, AZ USA; 9 Department of Ecology and Evolutionary Biology, University of Arizona, Tucson, AZ USA

**Keywords:** Tropical ecology, Microbial ecology

## Abstract

Understanding how species-rich communities persist is a foundational question in ecology. In tropical forests, tree diversity is structured by edaphic factors, climate, and biotic interactions, with seasonality playing an essential role at landscape scales: wetter and less seasonal forests typically harbor higher tree diversity than more seasonal forests. We posited that the abiotic factors shaping tree diversity extend to hyperdiverse symbionts in leaves—fungal endophytes—that influence plant health, function, and resilience to stress. Through surveys in forests across Panama that considered climate, seasonality, and covarying biotic factors, we demonstrate that endophyte richness varies negatively with temperature seasonality. Endophyte community structure and taxonomic composition reflect both temperature seasonality and climate (mean annual temperature and precipitation). Overall our findings highlight the vital role of climate-related factors in shaping the hyperdiversity of these important and little-known symbionts of the trees that, in turn, form the foundations of tropical forest biodiversity.

## Abstract (web summary)

Oita, Arnold and colleagues investigate the interplay between abiotic and biotic factors in lowland forest microbial communities. Their results indicate that endophyte richness negatively correlates with temperature seasonality. Both biotic and abiotic factors should be considered when studying endophyte community diversity drivers.

## Introduction

Understanding how species-rich communities persist has roots in ecological studies of biotic interactions and abiotic factors at local to landscape scales^1,2^. In highly diverse tropical forests, tree diversity is structured by the interplay of edaphic and climate characteristics, and by associated biotic interactions with dispersers and natural enemies such as herbivores and pathogenic fungi^3–7^. These factors come together to define broad patterns in the distribution of tropical tree communities at local to landscape scales, with classical work showing that climate (temperature and precipitation) and seasonality (intraannual shifts in temperature and precipitation) play an essential role: wetter and less seasonal tropical forests typically harbor higher tree density and diversity compared to drier and more seasonal tropical forests, partly because of intense pressure from natural enemies under stable and productive environmental conditions^3,8,9^. Similar patterns also are observed in plant-associated animals such as pollinators (e.g., hummingbirds and butterflies^10,11^), emphasizing the importance of climate, both in absolute terms and in terms of seasonality, as a key driver of biodiversity across not just the tropics but on a planetary scale (see also refs. ^12–14^).

Tropical forest trees support and interact with a tremendous diversity of associated organisms, with particularly high diversity occurring among the fungal symbionts that affiliate with living tissues such as leaves. These foliar endophytic fungi (hereafter, endophytes) are abundant and highly diverse in tropical forests, where they play important roles in protecting plants against pathogens^15^, altering leaf water relations^16^, and influencing leaf photosynthetic efficiency^17^. They typically are transferred horizontally as airborne spores and hyphae, forming communities that are structured at local scales by host phylogeny and traits such as secondary metabolites and leaf phenology^15,18–20^. At a larger scale, environmental factors such as temperature, precipitation, and vegetation are relevant to endophyte community structure in all biomes surveyed to date^21–24^, underscoring the interplay of abiotic and biotic drivers in shaping assemblages of these hyperdiverse and important symbionts.

Endophytes in tropical forests rarely demonstrate strict-sense host specificity, instead forming distinct communities in co-occurring host species^25,26^. However, recent studies argue that despite apparent host generalism in terms of affiliations, many fungi in tropical forests demonstrate strong functional specialization, interacting with particular host species, or with hosts that have particular traits, in distinctive and ecologically important ways^7^. Because reproductive propagules of endophytes are airborne, it is plausible that they may disperse across landscapes and thus traverse boundaries of proximate but distinct forest types, with little definition due to landscape-level gradients climate or seasonality. Alternatively, endophytes may demonstrate structure across tropical forests that can be traced directly to climate-related factors, consistent with the regional endemism and local uniqueness of plant communities that are defined by marked climate gradients in tropical regions^2,3,9^ and in line with the potential for local, functional specialization of endophyte assemblages under particular biotic or abiotic regimes^7,27^.

Evaluating these predictions requires landscape-scale surveys that take into account not only climate and seasonality, but also the many biotic factors that shift with these major drivers of biodiversity, including climate- and seasonality-related gradients of plant community composition, richness, and phylogenetic diversity; plant functional traits, such as chemical and physical defenses; and vegetation structure, which can influence the heterogeneity and suitability of local environments for fungal life cycles (e.g., ref. ^28^). More broadly, understanding the affiliations of endophytes for particular climate regimes is key to charting their diversity and roles in ecosystem services at a regional and global scale.

The rich history of studies on the diversity of tropical tree communities^1–9^ provides a basis for a series of predictions that, if sustained, would link climate, plant communities, and endophyte assemblages, with implications for high endemism of symbionts at small spatial scales and the potential for local functional specificity^27^. Specifically, if the factors shaping endophyte communities echo those shaping the communities of their host plants, we would predict that endophyte richness should follow well-documented patterns for tropical trees described by Givnish^3^: as for trees, endophyte richness should increase with mean annual precipitation (MAP) in lowland tropical forests (i.e., forests with elevation < 800–1000 m; typically taller, with higher aboveground biomass, and more diverse forests compared to montane forest) (Prediction 1); endophyte richness should decrease with increasing seasonality at a landscape scale, as for trees (Prediction 2); and endophyte communities should be structured at a landscape scale according to climate and seasonality (Prediction 3a), with high local endemism and turnover over sharp ecological gradients despite short geographic distances between forests (Prediction 3b).

**Fig. 1:**
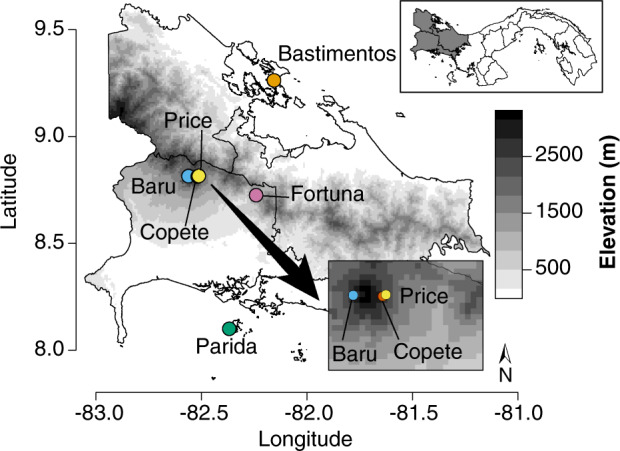
Sampling sites for collection of endophytic fungi from leaves of tropical trees in western Panama. Upper inset shows the focal region in Panama (shaded region), depicted in more detail in the main panel. The present study was conducted in Bocas del Toro (Bastimentos) and Chiriquí (all other sites), with the Pacific Ocean to the south and Caribbean Sea to the north. Shading corresponds to elevation. Lower inset clarifies relative locations of three proximate sites. Site and plot details are given in Table 1 and Supplementary Data 1. Map data were downloaded from the Database of Global Administrative Areas version 2.8. Values for elevation were retrieved from ref. ^44^.

We tested these predictions in the context of a global biodiversity hotspot, working at a landscape scale from the Pacific to the Caribbean side of the Isthmus of Panama. This area has high turnover in both climate (mean annual temperature (MAT) as a function of elevation; MAP as a function of the Pacific to Caribbean rainfall gradient) and seasonality (from strong dry seasons typical of the Pacific lowlands to everwet conditions typical of the Caribbean lowlands) over short geographic distances^25^. We collected fresh, apparently healthy leaves of angiosperm trees and shrubs in six primary forests in western Panama ranging from sea level to nearly 3000 m above sea level (masl) (Fig. 1 and Table 1). We focused on plant species that were representative of each forest type and locality^29,30^ with the expectation that few species would occur in multiple sites. We measured functional traits of leaves relevant to physical and chemical defense; characterized vegetation structure, plant community composition, plant species- and phylogenetic diversity, climate, and seasonality in 12 study plots; and used culture-free methods to sample endophyte communities. Our study encompasses over 2400 putative endophyte species to show that variation in endophyte communities across tropical forests mirrors closely the factors shaping the distributions and diversity of tropical forest trees, highlighting the vital role of climate and seasonality—both sensitive to anthropogenic effects at a global scale—in shaping the hyperdiversity of these important and little-known symbionts of plants, as well as the hosts they inhabit.

**Table 1:** Location, elevation, climate, and forest characteristics of plots surveyed for endophytic fungi in primary forests of western Panama, June–July 2016.

Site	Plot	Latitude (°)	Longitude (°)	Elevation (masl)	MAT (°C)	MAP (mm)	Temperature seasonality (SD × 100)	Precipitation seasonality (CV)	Canopy height (m)	Basal area (m^2^/ha)
Parida	Parida 1	8.097233	−82.366300	6	26.6	2693	732.8	67.0	35	33.3
Parida	Parida 2	8.097617	−82.365967	6.5	26.6	2693	733.0	67.0	30	19.5
Fortuna	Fortuna 1	8.722283	−82.238833	1213	20.1	2872	422.2	41.9	12	13.8
Fortuna	Fortuna 2	8.722333	−82.238817	1254	20.2	2873	422.2	41.9	11	14.9
Copete	Copete 1	8.809767	−82.515800	2902	11.6	2593	621.1	53.6	25	33.3
Copete	Copete 2	8.809717	−82.515050	2912	11.8	2583	623.1	53.7	30	35.6
Baru	Baru 1	8.811433	−82.559550	2436	12.3	2568	630.5	54.1	8	14.9
Baru	Baru 2	8.811150	−82.559417	2447	12.3	2570	630.1	54.1	5	25.3
Price	Price 1	8.812250	−82.51025	2532	12.8	2523	643.0	54.0	35	36.7
Price	Price 2	8.812017	−82.510317	2592	12.8	2524	642.4	54.0	35	32.1
Bastimentos	Bastimentos 1	9.258808	−82.155010	8	25.8	3154	596.0	28.0	25	14.9
Bastimentos	Bastimentos 2	9.258883	−82.154433	10	25.8	3154	596.0	28.0	25	17.2

Climate data were retrieved from ref. ^44^. For additional details regarding plant communities and plot characteristics see Supplementary Data 1. Plant identities and related information are shown in Supplementary Data 2.

*MAT* mean annual temperature, *MAP* mean annual precipitation, *SD* standard deviation, *CV* coefficient of variance.

## Results

**Fig. 2:**
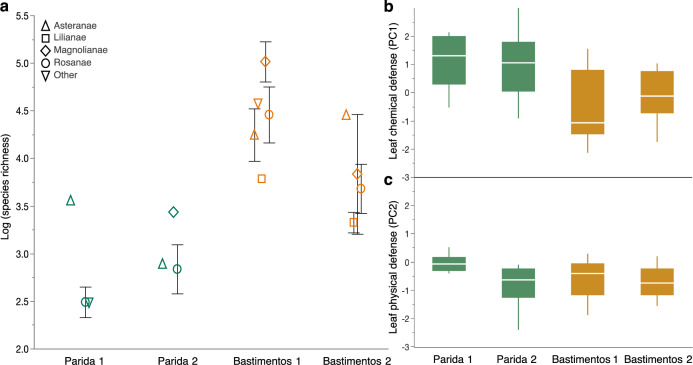
In leaves of representative angiosperms, endophyte richness was greater in the everwet, aseasonal lowland forest (Bastimentos) vs. the seasonally dry lowland forest (Parida), even when differences in leaf chemistry are considered. **a** Log species richness of endophytes for plants in each plot (*n* = 37 biologically independent samples), with shapes corresponding to superorders of host plants. For statistical analyses (see “Results”), plots were pooled within each site, variation due to leaf chemical defenses was accounted for, host species identity was treated as a random factor, and site was treated as a fixed effect. For a list of species corresponding to each plot and superorder, see Supplementary Data 2. Error bar represents standard errors. **b** Leaves of representative species at Parida had higher quantities of leaf chemical defenses than leaves of representative species at Bastimentos. PC1 reflects phenols, flavonoids, and condensed tannins (Supplementary Table 1). For statistical analyses, plots were pooled within sites and data were analyzed with species as a random factor and site as a fixed effect (see “Results” and Supplementary Data 2). Overall, representative leaves at Parida had higher flavonoid and tannin content relative to leaves of representative species at Bastimentos (Supplementary Data 2). **c** Leaf physical defenses (PC2, which largely reflects leaf mass per area; Supplementary Table 1) did not differ meaningfully between the two forests. Center line of box plot represents median; box limits, the upper and lower quartiles; whiskers, the 1.5× interquartile range.

We first tested the prediction that, as for tropical trees, endophyte richness should increase with MAP in lowland tropical forests (Prediction 1). We focused our collections at two extremes of the rainfall gradient that crosses the Isthmus of Panama: Isla Parida, off the Pacific coast, with a strongly seasonal pattern of rainfall and a marked dry season (hereafter, Parida); and Isla Bastimentos, off the Caribbean coast, with an aseasonal and markedly wetter climate (hereafter, Bastimentos; Fig. 1, Table 1, and Supplementary Data 1). The two islands host primary lowland forest characteristic of each region, with similar stem richness, density, and canopy height (Table 1 and Supplementary Data 1). Both islands are of similar area and are located a similar distance from the mainland (ca. 8 km; Fig. 1). In each site we contemporaneously surveyed endophytes associated with mature, healthy leaves of 8–10 species of representative angiosperms in each of two plots. We rarefied and log-transformed endophyte species richness for each plant (37 plant collections representing 26 species, 24 genera, 20 families, and 14 representative orders of angiosperms; Supplementary Data 2), verified that sampling was sufficient for intersite comparisons (Supplementary Fig. 1), and compared richness between the drier, more seasonal forest and the wetter, less seasonal forest, taking into account leaf defenses, which were predicted to differ between forests based on previous studies^3^. We defined leaf defenses in terms of two principal components that describe the majority of chemical defense (flavonoids, condensed tannins, and phenols) and physical defense (leaf mass per area (LMA)) (Supplementary Data 2, Supplementary Table 1, and Fig. 2).

We then examined endophytes in the most common superorder in samples from both sites (Rosanae: 21 collections representing 13 species in 13 genera, 11 families, and 5 orders) (Supplementary Data 2). Richness of endophytes in leaves of representative species did not associate meaningfully with the range of leaf defenses within each site (Supplementary Data 2). However, even though comparisons between the two sites revealed that leaves from both sites had similar levels of physical defense (*p* = 0.5517, with host species identity as a random factor), the two sites differed markedly in the degree to which leaves were defended chemically: leaves at Parida contained higher levels of chemical defense than those at Bastimentos (*p* = 0.0461, with species identity as a random factor; Supplementary Data 2). The same pattern was observed when all plants surveyed in both locations were considered (Asteranae, Caryophyllanae, Lilianae, Magnolianae, Rosanae, and Santalanae): leaves were better defended in terms of chemistry in the seasonal, drier forest at Parida than in the aseasonal, wetter forest of Bastimentos (*p* = 0.0079, with species identity as a random factor; see Fig. 2). In general, leaves at Parida were characterized by higher flavonoid and condensed tannin content than those from Bastimentos (*p* = 0.0271 and *p* = 0.0084, respectively, with species identity as a random factor) (Supplementary Data 2).

Therefore, to determine the degree to which climate-related factors could explain differences in endophyte richness between these forests, we first accounted for variation in endophyte richness due to chemical defenses of leaves, and then examined the residuals from that analysis. When variation due to leaf defenses was accounted for, endophyte richness was significantly higher in the less seasonal, wetter forest (Bastimentos), both overall and in the best-represented superorder (*p* = 0.0009 and *p* = 0.0027, respectively, with host species identity as a random factor) (Fig. 2).

Although consistent with Prediction 1, this result could not be dissected to differentiate effects of climate factors (MAT and especially MAP, Table 1) vs. seasonality (temperature- and especially precipitation seasonality, Table 1), as Parida is both drier and more seasonal, and Bastimentos is both wetter and less seasonal. Therefore, we evaluated richness of endophytes across two plots in each of six sites ranging from lowland to montane forests across the Isthmus of Panama (Fig. 1, Table 1, and Supplementary Data 1 and 2), with models attentive to factors that vary across forests as a function of elevation (seasonality; climate; vegetation structure, which included host phylogenetic diversity and stem richness on plots, among other factors; host factors including leaf defenses and other traits; and spatial factors that account for the uniqueness of each forest type; see Supplementary Methods, Supplementary Tables 2 and 3 for details). With these analyses we tested the prediction that, as for tropical trees, endophyte richness should decrease with increasing seasonality at a landscape scale (Prediction 2).

**Fig. 3:**
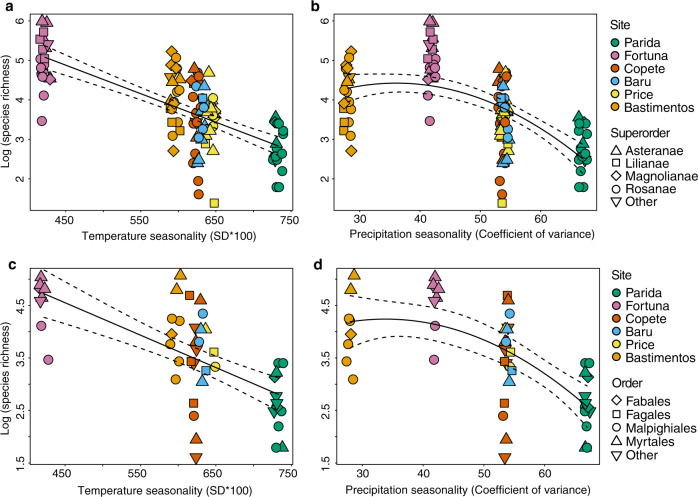
Species richness of endophytes in tropical trees reflects both temperature seasonality and precipitation seasonality. Richness decreased (**a**) linearly as temperature seasonality increased, and (**b**) as a quadratic function with respect to precipitation seasonality (*R*^2^ = 0.48 and 0.36, respectively; *p* < 0.0001 in each case, *n* = 106 biologically independent samples). Colors correspond to sites (Fig. 1) and shapes correspond to superorders (Supplementary Data 2). The same pattern was observed in endophyte richness in the Rosanae, the most frequently collected superorder of host plants (**c**, **d**: *R*^2^ = 0.43 and 0.42, respectively, and *p* < 0.05 in each case; shapes correspond to orders, Supplementary Data 2). Although additional factors may covary with seasonality (e.g., factors in Supplementary Table 3), they have little explanatory power after temperature and precipitation seasonality are considered (Fig. 4). Dotted lines represent 95% confidence interval.

**Fig. 4:**
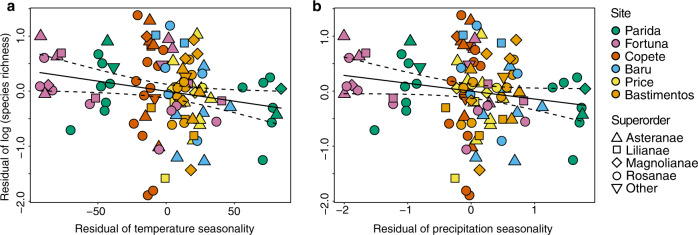
Relationships of endophyte richness to seasonality remained robust when spatial factors, climate, vegetation, and host factors were considered. Residual of species richness of endophytes in all hosts as a function of the residual of temperature seasonality (**a**) and residual of precipitation seasonality (**b**) after accounting for spatial factors, climate, vegetation, and host factors (**a**: *R*^2^ = 0.03, *p* = 0.031; **b**: strongly approaching significance; *p* = 0.067, *n* = 106 biologically independent samples). These factors are described in full in “Methods” and Supplementary Methods. We illustrate the direct relationship of endophyte richness with temperature seasonality and precipitation seasonality in Fig. 3. Dotted lines represent 95% confidence interval.

Consistent with Prediction 2, data from 12 plots and a total of 106 individual plants representing 26 orders, 43 families, and 74 species of angiosperms (Supplementary Data 2) showed that overall endophyte richness was associated negatively with seasonality in terms of seasonal variation in temperature and precipitation (Fig. 3, *p* < 0.0001 in each case). These relationships remained robust when spatial factors, climate, vegetation, and host factors were considered simultaneously (Fig. 4, *R*^2^ = 0.03, *p* = 0.031; for components of each factor, including plant diversity measures, see Supplementary Methods).

Based on these results, we predicted that as for communities of tropical trees, endophyte community structure should reflect climate and seasonality at a landscape scale (Prediction 3a). We first examined the mock community data (i.e., positive controls with 31 phylogenetically diverse fungi with known DNA concentration for evaluation of sequencing quality; see “Methods” and Supplementary Methods) to confirm that expected and observed read numbers were positively associated for Ascomycota (Supplementary Fig. 2), which accounted for 75% of reads for our collections after quality control. The positive association observed in that analysis led us to consider not only presence–absence data in quantifying community composition but also relative abundance values (Supplementary Fig. 2 and Supplementary Methods).

**Fig. 5:**
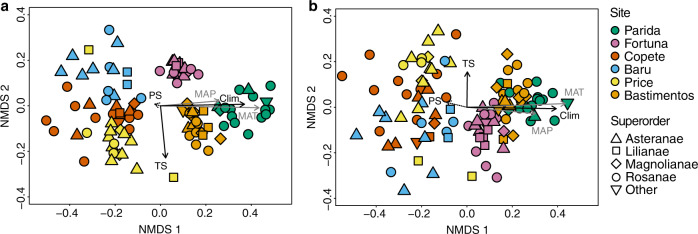
Endophyte community composition differed as a function of climate (MAT and MAP) and temperature seasonality. When evaluated on the basis of (**a**) presence–absence data (Jaccard index) and (**b**) relative abundance data (Morisita index) for all hosts plants surveyed (*n* = 106 biologically independent samples). For analyses of the most frequently sampled superorder (Rosanae) see Supplementary Fig. 3. Each dot represents an endophyte community from an individual plant. Colors correspond to sites and shapes correspond to superorders of host plants. Black arrows represent the significant vectors of climate PC1 (Clim, considering MAT and MAP) and temperature seasonality (TS) fitted to the ordination scale (**a**: *R*^2^ = 0.61 and 0.23, respectively, *p* < 0.01 in each case; **b**: *R*^2^ = 0.69 and 0.11, respectively, *p* < 0.01 in each case). Gray arrows represent the vector of each climate factor (MAT and MAP) (**a**: *R*^2^ = 0.83 and 0.30; **b**: *R*^2^ = 0.84 and 0.41, respectively; *p* < 0.001 in each case). The dotted arrow represents the vector of precipitation seasonality (PS, *p* > 0.1 in each case). Additional factors also characterize each site (Supplementary Table 3) and have measurable impacts on endophyte community structure, but the signatures of climate and temperature seasonality most effectively define the ordination space for endophytes at a landscape scale.

Ordination of endophyte communities revealed robust clustering of endophyte communities by site, with strong signatures of climate (MAT and MAP) and temperature seasonality in defining endophyte communities (all host plants, Fig. 5; Rosanae, Supplementary Fig. 3). Variation partitioning across the entire data set highlighted contributions of diverse factors that characterize each site in shaping endophyte communities, with the strong signature of climate and seasonality reflected clearly at a landscape scale (Supplementary Table 3).

The clustering of endophyte communities by site reflects a prevalence of distinctive operational taxonomic units (OTUs) in each study area, consistent with Prediction 3b: on average 60.0% of OTUs found in each site were unique to that site alone (Supplementary Table 4), despite small geographic distances among sites. The average intersite distance was 54.1 km (95% CI, 34.4–73.9 km), with the most distant sites located only 131.2 km apart (Fig. 1 and Table 1). Even sites located ≤5.4 km from one another (Fig. 1; intersite distances 0.7–5.4 km) differed markedly in their endophyte communities, with 51.6–52.8% of OTUs found in Baru, Price, and Copete unique to each of those locations (Supplementary Table 4) despite thorough sampling of local endophyte richness (Supplementary Fig. 1). These sites are similar in terms of climate and precipitation seasonality, but differ in temperature seasonality (Table 1, see also Fig. 5 and Supplementary Fig. 3).

The same major lineages (classes) of Ascomycota were observed in all sites, suggesting that most of the locally distinct endophytes are distinct haplotypes, species, or genera of fungi, rather than taxa in higher rank. Consistent with previous studies^25^, we observed a marked difference in the prevalence of focal classes (Dothideomycetes, Eurotiomycetes, Leotiomycetes, Sordariomycetes) that we now can interpret through the lenses of climate and seasonality (Supplementary Fig. 4). Sordariomycetes decreased in prevalence as temperature seasonality increased (*R*^2^ = 0.14, *p* < 0.0001), consistent with the observation that they often are extremely common as endophytes in everwet tropical forests^20,25^. Common orders of Sordariomycetes identified with relatively high certainty (see Supplementary Data 2) included Glomerellales, Hypocreales, and Xylariales. In contrast, Dothideomycetes increased in prevalence over that same gradient (*R*^2^ = 0.12, *p* = 0.0004; Supplementary Fig. 4 and Supplementary Data 3). The hyphae of many Dothideomycetes isolated in previous culture-based studies of endophytes from strongly seasonal lowland forests are robustly pigmented, consistent with seasonal increases in UV radiation in such locations^23,25,26,28,31,32^. Such pigmentation was common among the most prevalent orders of Dothideomycetes identified with relatively high certainty (Botryosphaeriales, Pleosporales, and Capnodiales). In previous studies of tropical endophytes, Leotiomycetes have been considered rare^25,31^, yet we detected them relatively frequently in the oak-dominated, high-elevation forests that share major lineages of plants with the strongly seasonal forests of the temperate zone, where global studies indicate that Leotiomycetes often are more prevalent^25,31,32^.

## Discussion

The highly diverse plant communities in tropical forests support diverse assemblages of associated organisms whose distributions may be predicted to track those of the plants with which they affiliate. Classical work in plant ecology^1–9^ has revealed a suite of patterns with regard to gradients of diversity in tropical trees. Here we extend this framework to hyperdiverse foliar endophytes that occur within living leaves, where they impact plant physiology, plant health, plant response to stress, and ultimately, ecosystem services of Earth’s most species-rich plant communities^15–17^.

By extending the predictive framework for plants to foliar symbionts, we found that, as for tropical trees, species richness of tropical endophytes increased with increasing precipitation in lowland forests and decreased with seasonality at a landscape level. Moreover, we found that endophyte communities were structured at a landscape scale according to climate and temperature seasonality, with high local endemism over climate-defined gradients despite small geographic distances between sampling sites.

Overall, our data reveal signatures of seasonality of temperature and precipitation in defining endophyte richness; climate (defined by MAT and MAP) and temperature seasonality in defining endophyte community structure and local uniqueness of endophytes in geographically proximate forests; and temperature seasonality with respect to the relative prevalence of major lineages of Ascomycota across a landscape of diverse forests that also shift in the relative abundance of major plant clades. Our inclusion of local plant diversity in our analyses (a component of the vegetation factor, Supplementary Methods) reveals a signature of climate factors in endophyte richness and composition that cannot be attributed only to vegetation and associated plant diversity. Additionally, previous studies showed no evidence of year-to-year or month-to-month variation in foliar endophyte richness and community structure in tropical forests, which indicates that our surveys were representative of the typical endophytes in each site^20,33^. Taken together, these analyses suggest that as for trees, endophytes will be sensitive to shifts in climate in tropical regions, which can include changes not only in temperatures and especially the absolute quantity of precipitation, but also changes in the seasonality of rainfall and temperature.

The relationship between endophyte richness and seasonality corresponds with the growing appreciation of climate and its intraannual dynamics as a key driver of biodiversity at a global scale, important not only for tropical trees but also for the symbiotrophic and saprotrophic fungi that interact with plants in all terrestrial ecosystems^13,14,21,22^. In a global study of soil fungi, Tedersoo et al.^12^ showed that the richness of two classes of soil fungi that are also abundant as endophytes, Sordariomycetes and Eurotiomycetes, correlates negatively with seasonality. That study also found that the composition of plant-pathogenic and saprotrophic fungal communities in soil was related robustly to seasonality at a global scale. Many endophytes are closely related to pathogens and saprotrophs or are thought to have pathogenic or saprotrophic free-living life stages^31^, suggesting that at the landscape scale, similar seasonality-mediated drivers are important for endophytes in the tropics and beyond (see also ref. ^27^).

**Fig. 6:**
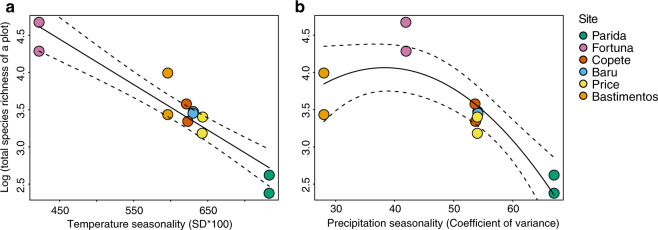
The size of the local species pool of endophytes reflects seasonality. As for endophyte richness in host plants (Fig. 3), we observed lower total species richness in each plot as (**a**) temperature seasonality and (**b**) precipitation seasonality increased (*R*^2^ = 0.88 and 0.77, respectively; *p* < 0.0001 in each case). We used total number of endophyte OTUs found at each plot as a proxy of local species pool size. We calculated species richness of each plot as the number of OTUs found at each plot, and normalized by the number of samples to account for differences in sample size among plots. Dotted lines represent 95% confidence interval.

Endophyte communities may be shaped by climate and seasonality in diverse ways. For instance, strong dry seasons could impose a strong physiological filter on these horizontally transmitted fungi (which must exist outside of leaves for parts of their life cycle^28^), potentially leading to lower richness of the local species pool of endophytes. This is consistent with the association between seasonality and endophyte richness after accounting for vegetation and associated host factors (Fig. 4) and with our observations of lower numbers of total endophyte species as a proxy of local species pool in more seasonal forests (Fig. 6 and Supplementary Table 4). Thus, much like other organisms that rely on leaves, it appears that endophytes are limited by the robust physiological filter of strong dry seasons in the tropics. This interpretation is consistent with experiments in a seasonal forest at Barro Colorado Island, Panama, where survival of endophyte propagules and resulting colonization of leaves, was limited by the high temperatures, lower humidity, and/or UV irradiance typical of the dry season^28^.

Stronger seasonality also is thought to limit the number of woody species in tropical forests, in part by restricting feedbacks from seasonally limited natural enemies^3,9,34,35^. Natural enemies such as specialized herbivorous insects and pathogens selectively reduce survival of host plants when densities of conspecifics are high at a local scale. This facilitates recruitment of heterospecific plant species and leads to the increase of plant richness at the community level^3,7–9^. In more seasonal forests, the facilitation of heterospecific recruitment is thought to be less intense because seasonal changes decrease activity of natural enemies compared to aseasonal tropical forests^9^. Such a decreased species richness of host plants could affect endophyte diversification in situ, potentially limiting the richness of the local symbiotic species pool. However, we could not test the indirect effect of seasonality on the local species pool in terms of the phylogenetic and species richness of host plants due to the collinearity among these variables with geographic distances.

**Fig. 7:**
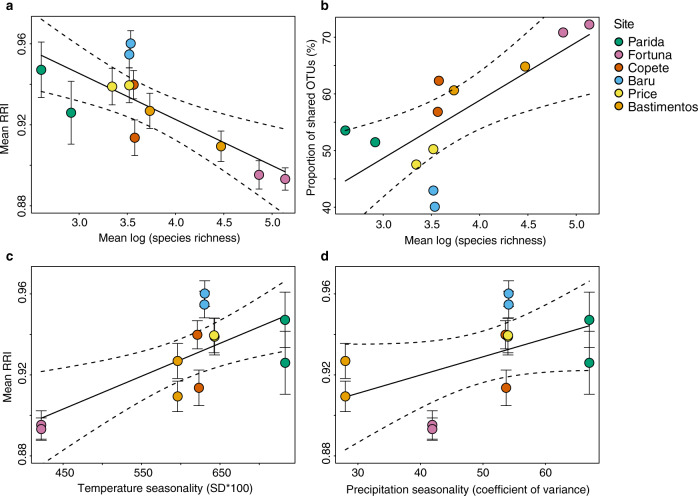
Perspectives on host generalism, calculated with two metrics. **a** The average number of observed plant species with which OTUs affiliate in each plot was calculated from occurrence data and calculated as resource range index (RRI, see “Methods”; higher RRI represents a lower number of hosts per OTU). Endophyte richness was negatively associated with RRI (i.e., in plots with higher endophyte richness, the number of hosts used per OTU was higher) (*R*^2^ = 0.55, *p* = 0.004). **b** The proportion of shared OTUs was calculated as the proportion of OTUs that occurred in more than one plant species, divided by the total number of OTUs in the plot. Consistent with **a**, we found that as richness increased, the number of nonspecialists increased (*R*^2^ = 0.47, *p* = 0.009). We then considered the relationship of the RRI metric to seasonality, finding that (**c**) temperature seasonality and (**d**) precipitation seasonality could explain variation in RRI (*R*^2^ = 0.47, *p* = 0.009; *R*^2^ = 0.20, *p* = 0.08, respectively). Error bar represents standard errors. Dotted lines represent 95% confidence interval.

A complementary perspective emerges when we consider host ranges of endophytes. It is widely recognized that the relatively consistent rainfall and temperature typical of aseasonal tropical forests can allow specialization by some organisms on relatively predictable resources (e.g., photosynthates^36^). However, high diversity of trees in less seasonal forests may preclude specialization due to the challenge of locating compatible host species in a backdrop of highly diverse host communities, leading to wider host ranges in some taxa that use plant tissues (e.g., herbivorous insects^37^). In this scenario, we would expect increased generalism of endophytes in less seasonal forests with richer plant communities, which is what we observed here (Fig. 7; e.g., Fortuna, with a highly diverse tree community at local scales; see also refs. ^38,39^).

**Fig. 8:**
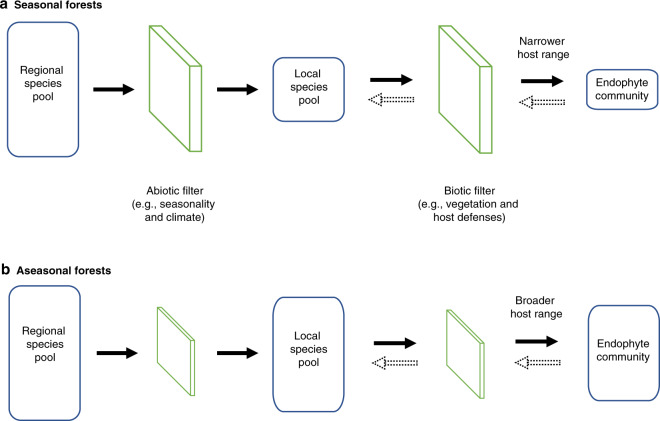
Conceptual diagram linking abiotic and biotic filters from the regional endophyte species pool to endophyte richness in host plants. Here, abiotic and biotic filters sieve the regional species pool (far left) and local species pools (middle) toward endophyte richness in host plants (far right), as proposed for (**a**) seasonal forests and (**b**) aseasonal forests (see “Discussion”). The regional species pool is defined here as the pool of endophyte species that is physically dispersible to each locality. The local species pool contains species that appear locally (i.e., at each plot, in the work presented here) after being filtered by abiotic factors such as seasonality and other climatic factors at each plot. The local species pool then is filtered further by biotic filters including vegetation or host factors, which shape the endophyte community detected in each host plant (rightmost blue rectangle). The rectangle representing each species pool or endophyte community is sized to reflect the number of species. Dotted lines represent potential feedback (e.g., the feedback of vegetation to local species pool, the feedback of endophyte interactions influence host defenses) that cannot be tested in this study. We suggest that aseasonal forests impose a weaker physiological filter on these horizontally transmitted fungi, which could lead to higher richness of the local species pool of endophytes as a direct effect (Fig. 6). As an indirect effect, high diversity of trees in aseasonal forests may preclude specialization due to the challenge of locating compatible host species in a backdrop of highly diverse host communities, leading to wider host ranges (Fig. 7).

In turn, the more robust chemical defenses in leaves in seasonal vs. aseasonal forests^37^ (see also Fig. 2) also may be important in filtering the local pool of fungal species, leading to relatively narrow host ranges and lower species richness in forests with pronounced dry seasons. In such a scenario we would expect more marked differences in leaf chemistry among species (as in ref. ^28^) in seasonal forests vs. aseasonal forests. When we examined chemical defenses in our lowland plots, flavonoids and phenolics did not differ among species (Supplementary Data 2). However, among species variation in total condensed tannins was greater in plots in seasonal lowland forest (Parida) than aseasonal lowland forest (Bastimentos) (Supplementary Data 2). In addition to examining qualitative aspects of leaf chemical defenses, and components such as volatiles^40^ or available carbon and nitrogen^26,41^, future work should consider the relevance of these components in shaping endophyte communities: tannins are climate sensitive^42^ and may be important in influencing the host range and richness of tropical endophyte communities at multiple scales. Thus, endophyte richness may be shaped by climate and seasonality both directly and indirectly via host plants (Fig. 8).

Endophytes often are invoked as potentially extending the defensive phenotypes of the leaves they inhabit, and it is plausible that specific endophytes are recruited by specific hosts because of such a benefit^15^. Given the greater degree of chemical defense in leaves in more seasonal forests, and the observation of narrower host ranges among endophytes there, we would expect endophytes from seasonal forests to demonstrate greater bioactivity (i.e., capacity to produce secondary metabolites) relative to endophytes from aseasonal forests. However, previous work^43^ found that endophytes of aseasonal, cooler, and wetter forests in Panama were markedly more bioactive than their relatives in strongly seasonal lowland forests. It is possible that endophyte communities in seasonal forests are shaped more by abiotic filters and host chemistry, and endophytes of aseasonal forests—where richness of endophytes is very high—may be shaped more by interspecific competition, consistent with an antipathogen role of endophytes in certain tropical forest species, especially when under wet and humid conditions^15^. More generally, potential roles of endophytes in modulating chemical phenotypes of leaves, relevant for protecting leaves against biotic and abiotic stress under diverse climate conditions, merit further attention.

Distributions of tropical trees are shaped directly and indirectly by climate and seasonality^1–9^. Here, we considered the relevance of climate and seasonality for endophyte communities in leaves, while addressing factors that vary with these factors at a landscape scale. By considering leaf defenses, vegetation structure, host factors, and distinctive features of each site, we contextualize the relevance of climate and seasonality against the complex ecological backdrop characteristic of tropical regions. We note that all aspects we measured—from endophyte richness and community structure to leaf chemistry and physical defense, forest structure, and host factors—can be considered sensitive to shifts in climate and seasonality over time. In our survey across western Panama, we collected most plant species (66.7%) only once, consistent with marked turnover in plant communities as we moved from the lowlands of the Pacific coast to the lowlands on the Caribbean side ca. 131 km away. Such local uniqueness of plants thus extends to the many organisms that interact with them, including fungal endophytes. By showing that factors shaping endophyte communities across tropical forests mirror those shaping the distributions and diversity of tropical forest trees, we highlight the vital role of climate and seasonality in shaping the hyperdiversity of these little-known symbionts of plants. Foliar fungal symbionts play key roles in regulating plant health and productivity in tropical forests, such that understanding the direct and indirect impacts of shifts in climate and seasonality on endophytes, and their hosts, is fundamental to the future of tropical plant communities in a rapidly changing world.

## Methods

In June to July 2016, we collected leaves of mature, apparently healthy angiosperm trees and shrubs in six sites from lowland- to montane forests in western Panama (Fig. 1, Table 1, and Supplementary Data 1). Each site consisted of undisturbed primary forest (Supplementary Data 1). We focused only on mature, non-senescing, fully expanded leaves to reduce variation due to age of tissue. We generally worked on plants with accessible branches, but in closed forest many of those are very slow growing. We anticipate that all plants were multiple years old, and that there was no systemic bias in our sampling across sites. Leaves were considered healthy if they showed no visible signs or symptoms of disease. Plants were considered healthy if the overall aspect of the plant revealed little evidence of disease, as measured on an index from low (few leaves with symptoms of any kind; no evident dead branches or leaves; no evident wood rot) to high (high scores on each measure).

In each site we established two 4 × 5 m plots ca. 30 m apart. For each plot we recorded elevation, forest condition and type, and environmental and forest characteristics (Table 1 and Supplementary Data 1). We obtained climate data for each plot from the WorldClim database^44^ (Version 1.4, http://www.worldclim.org/bioclim.htm, accessed in May 2018, with the finest resolution available, 0.5 min). We characterized vegetation type following ref. ^45^. Published estimates of tree species richness for each forest type were not available for most sites due to a lack of forest plots and extensive characterization of tree communities. The exception was the Fortuna area, recognized for its high species richness^46^. Our remaining sites were chosen to represent major forest types from the Pacific to the Caribbean, spanning a known range of climate and seasonality factors (Table 1), land use history^47^, first-hand observations of forest composition (Ibáñez, unpubl. data), and estimates of plant species richness based on^48^. Broadly, we anticipated similar tree species richness in Bastimentos to that of the well-characterized forests at the Caribbean end of the transisthmian gradient of the Panama Canal watershed^48,49^; in Parida, similar to that of the Pacific side of the Canal^49^; and lower diversity in the montane forests of Baru, Price, and Copete, consistent with the decline tree species richness that occurs at higher elevations in the region^50^.

In each plot we collected five mature, healthy leaves from one representative of each of ten species of angiosperms (i.e., 120 individual plants in total, representing 27 orders, 48 families, and 79 species; 106 were ultimately included in our analyses) (Supplementary Data 2). We collected species that were representative of each forest type and locality^29,30^ with the expectation that few species would occur in multiple sites because of high local turnover in species composition at a landscape scale (see Supplementary Methods). We stored fresh leaves in plastic bags at 4 °C for ≤72 h prior to processing for analyses of leaf defenses, at which point we also set aside fresh tissue for DNA extraction. For processing, we cut each fresh leaf into half along the midvein. From one half of each fresh leaf we immediately collected five leaf discs (each 0.32 cm in diameter), which we used to measure LMA. We used the remainder of that half to measure total phenolics, total flavonoid, and condensed tannin (see Supplementary Methods).

### DNA extraction and ITS rDNA sequencing

We cut the remaining half of each fresh leaf into 1 × 2 mm segments for endophyte analysis. We pooled segments for each individual and surface sterilized them by agitating sequentially in 95% EtOH for 10 s, 0.5% NaOCl for 2 min, and 70% EtOH for 2 min^51^. We placed 96 segments into CTAB buffer (24 segments per mL of CTAB buffer^52^) and stored the samples at −80 °C prior to DNA extraction. We used the Qiagen PowerPlant Pro-htp 96 Well kit (Qiagen, Valencia, CA, USA) to extract total genomic DNA from each set of leaf segments (i.e., four extractions per individual plant^53^). Details of DNA extraction are described in the Supplementary Methods.

We used a two-step PCR approach for fungal ITS rDNA amplicon sequencing on the Illumina MiSeq platform (Illumina, Inc., San Diego, CA, USA) with primers ITS1F and ITS4 following^54^ with a summary presented in the Supplementary Methods. We pooled 20 ng of amplified DNA with barcoded adapters (IBEST Genomics Resource Core, Moscow, ID, USA) from each sample into a single tube for sequencing on the Illumina MiSeq (300-basepair paired-end sequencing) by the University of Idaho IBEST Genomics Resources Core. We sequenced negative controls and two mock communities as positive controls with our samples (Supplementary Data 4 and Supplementary Methods).

We performed demultiplexing, quality control, and dereplicating as described in the Supplementary Methods. We used forward reads (i.e., ITS1) in our analyses because the forward reads had higher quality than the reverse reads (see Supplementary Methods). We excluded 14 samples with fewer than 1000 reads for further analyses. Dereplicated sequences were clustered into OTUs at 95% sequence similarity, consistent with previous work on endophytic Ascomycota^18,32,52^. We used 95% as clustering threshold over zero-radius OTUs (i.e., zOTUs, also known as amplicon sequence variants^55^) because we confirmed that the number of zOTUs in the mock communities was inflated at that stringency (48 zOTUs were found in mock communities, but 31 species were included per ref. ^54^; this was resolved by using the 95% similarity cutoff). Processes of taxonomic assignment and rarefaction are described in Supplementary Methods.

### Statistics and reproducibility

We used principal component analysis to define chemical defenses and physical defense of leaves (PC1 and PC2, respectively; Supplementary Data 2 and Supplementary Table 1, Supplementary Methods). We evaluated leaf defenses for plants from Parida and Bastimentos as described in Supplementary Data 2, and then used residuals from the regression of PC1 of leaf defenses to compare log-transformed richness of endophytes between these two lowland forests with different seasonality and climate.

To examine the relationship of seasonality and endophyte richness after accounting for other host- and environmental factors, we conducted variation partitioning based on partial linear regression analysis with endophyte OTU richness as a response variable and five explanatory variables, including seasonality and four covariates (spatial factor, climate factor, vegetation factor, and host factor). These explanatory variables are defined in full in the Supplementary Methods. We used PCA to summarize climate factors (MAP and MAT) to avoid multicollinearity. We used the first PC, which explained 87.5% of total variation in MAP and MAT, to represent a climate factor (Supplementary Table 2). Details of other factors (i.e., spatial factor, vegetation factor, and host factor) are described in the Supplementary Methods.

We visualized endophyte community structure via nonmetric multidimensional scaling. We used seasonality as a combined factor including two vectors, temperature seasonality and precipitation seasonality (see Table 1). We used the R package *vegan*^56^ version 2.5-2 for permutational analyses of variance to examine how endophyte community composition and structure differed as a function of seasonality and climate (Fig. 5 and Supplementary Fig. 3), and for variation partitioning based on partial distance-based redundancy analysis to calculate the variation in endophyte communities that was explained by seasonality and climate (Supplementary Table 3). We used the Jaccard index to measure the similarity of community composition based on presence–absence data, and the Morisita index to measure the similarity of community structure based on abundance data. All variables were calculated in *vegan*^56^. We used the same explanatory variables that we used for the species richness analysis in this analysis.

The number of observed plant species of each OTU was calculated as the resource range index (RRI^57,58^ that represents the number of observed host species normalized by the number of unoccupied plant species. RRI varies from 0 (found in all plants) to 1 (found in only one plant).

### Reporting summary

Further information on research design is available in the Nature Research Reporting Summary linked to this article.

## Supplementary information

Supplementary Information

Description of Additional Supplementary Files

Supplementary Data 1

Supplementary Data 2

Supplementary Data 3

Supplementary Data 4

Reporting Summary

## Data Availability

Sequences used in this study are deposited in NCBI Sequence Read Archive (SRA) under BioSample accession ID SAMN15675876-SAMN15675983 of SRA accession PRJNA649645.

## Code availability

All metadata and codes used in this study are publicly available in figshare (see ref. ^59^).
