# Supplementary material for: Climate and seasonality drive the richness and composition of tropical fungal endophytes at a landscape scale
Source: Commun Biol. 2021 Mar 9;4:313. doi: 10.1038/s42003-021-01826-7 (PMC7943826; doi:10.1038/s42003-021-01826-7)
Supplement: Supplementary file 1 — Supplementary Information [file 42003_2021_1826_MOESM1_ESM.pdf]

Supplementary information for

Climate and seasonality drive the richness and composition of tropical fungal endophytes at a landscape scale

Shuzo Oita[1], Alicia Ibáñez[2], François Lutzoni[3], Jolanta Miadlikowska[3], József Geml[4], Louise A. Lewis[5], Erik F. Y. Hom[6], Ignazio Carbone[7], Jana M. U'Ren[8], A. Elizabeth Arnold[1,9]

[1] School of Plant Sciences, University of Arizona, Tucson, AZ 85721, USA. [2] Independent researcher, Jadwin Av. 108B, Gamboa, Republic of Panama. [3] Department of Biology, Duke University, Durham, NC 27708, USA. [4] MTA-EKE Lendület Environmental Microbiome Research Group, Eszterházy Károly University, Leányka u. 6, H-3300 Eger, Hungary. [5] Department of Ecology and Evolutionary Biology, University of Connecticut, Storrs, CT 06369-3043, USA. [6] Department of Biology, Center for Biodiversity & Conservation Research, University of Mississippi, University, MS 38677, USA. [7] Center for Integrated Fungal Research, Department of Entomology and Plant Pathology, North Carolina State University, Raleigh, NC 27695, USA. [8] Department of Biosystems Engineering and BIO5 Institute, University of Arizona, Tucson, AZ 85721, USA. [9] Department of Ecology and Evolutionary Biology, University of Arizona, Tucson, AZ 85721, USA

Supplementary Methods

Sampling

In each plot we collected five mature, healthy leaves from one representative of each of 10 species of angiosperms (i.e., 120 individual plants in total, representing 27 orders, 48 families and 79 species) (Supplementary Data 2). Overall, each plant species was collected an average of 1.4 times, with the majority of plant species collected fewer than twice across our 12 plots (Supplementary Data 2). Vouchers of all host plants were deposited in the Herbarium of the University of Panama (PMA) with duplicates at the University of Arizona (ARIZ).

Analysis of leaf defenses

We cut each fresh leaf into half along the midvein. From one half of each fresh leaf we immediately collected five leaf discs (each 0.32 cm in diameter), which we used to measure leaf mass per area (LMA)[1]. We dried the remainder of that half at 40 ˚C[2]. We extracted 2.5 mg of the dried leaf tissue with 70% acetone for 2 h at 4 ˚C. We measured phenolics by the Folin-Ciocalteu method[3] with minor modifications. Briefly, we mixed 100 µl of leaf extract or gallic acid (standard) with 200 µl of 10% (v/v) Folin-Ciocalteu reagent. We added 800 µl of 700 mM Na_2CO_3, incubated the mixture at ca. 22°C for 2 h, and determined absorbance at 765 nm. We measured total flavonoids with (+)-catechin as a standard as described in ref. [4]. We measured the relative concentration of condensed tannins by the butanol-HCl method[5,6]. There is no suitable standard for the butanol-HCl method[6,7]. Therefore, we used an extract of *Ouratea lucens* from Parida 2 (Supplementary Data 2) as an internal standard because this species showed the highest concentration in our preliminary analysis. We averaged each measure (LMA, total phenolics, total flavonoids, and relative concentration of condensed tannins) for five leaves per individual to obtain an individual-level value for each defense trait (Supplementary Data 2).

DNA extraction and sequencing

To extract DNA we decanted the CTAB from each tube and lyophilized each set of leaf segments for 48 h. We added 0.6 g of pre-sterilized stainless steel beads to each tube (bead diameter 0.9-2.0 mm, 0.2 g; 3.2 mm, 0.4 g) and homogenized the segments for 45 s via bead-beating in a FastPrep-24 Tissue and Cell Homogenizer (MP Biomedicals, Solon, OH, USA). We used the Qiagen PowerPlant Pro-htp 96 Well kit (Qiagen, Valencia, CA, USA) to extract total genomic DNA from each set of leaf segments (i.e., four extractions per individual plant[8]). We pooled DNA extractions for each individual before amplification. We quantified the DNA concentration of each sample with the PicoGreen assay[9] (Supplementary Data 2). DNA samples and additional tissue samples in CTAB are archived at the University of Arizona.

We first amplified fungal ITS rDNA with primers ITS1F and ITS4, which were modified with universal consensus sequences CS1 and CS2 and 0-5 bp for phase-shifting (Integrated DNA Technologies Inc., Skokie, IL, USA)[10]. Each sample was amplified in three parallel reactions that each contained 1-2 µl of DNA template[11]. The product of each reaction was visualized with SYBR Green 1 (Invitrogen, Carlsbad, CA, USA) on a 2% agarose gel. We pooled the three PCR products per individual and diluted the pooled amplicons as needed with sterile, molecular-grade water (Thermo Scientific) to 1:5 based on band intensity. We used the diluted products (PCR1) as templates for a second PCR (PCR2) with barcoded adapters[11] (IBEST Genomics Resource Core, Moscow, ID, USA). We confirmed that primer dimers were minimized, quantified the products as above.

Negative controls for molecular analyses

We prepared PCR1 in a sterile, dedicated "pre-PCR" hood with sterile equipment and dedicated pipettes as described in ref. [11]. We pooled negative controls from DNA extractions (kit reagents) and PCR1 separately and used them as templates for PCR2 to ensure no contamination prior to PCR2. Although no contamination was detected, we prepared 5µl of each negative control as above and sequenced the negative controls in parallel with our samples.

Positive controls for molecular analyses

We sequenced two mock communities consisting of DNA from a known set of fungi as detailed in ref. [11]. The mock communities included 31 phylogenetically diverse fungi representing the major fungal taxa[12]. One community consisted of all 31 species represented by equimolar amounts of DNA (i.e., the 'even' mock community). The even mock community was used to evaluate potential primer- or sequencing bias[13]. The second community comprised DNA from the same fungal taxa in tiered concentrations ranging from 0.94 to 23.6 ng/µl based on the order of abundant phyla as endophytes ('tiered' mock)[14]. This mock community was used to evaluate whether read abundance after sequencing represented abundance in the original sample. We amplified 2 µl of each mock community in triplicate for PCR1 as described above, pooled the PCR1 products for each community, and diluted the products 1:5 with sterile, molecular-grade water. We used 1 µl of each diluted product as template for PCR2. Our analyses of the mock communities revealed that our approaches could capture phylogenetically diverse fungi and that the read abundance of each sample in the tiered community was positively correlated with the expected read number from each species (Supplementary Fig. 2, Supplementary Data 4). Therefore we used both abundance and presence-absence data in analyses of endophyte communities.

Post-sequencing quality control

We performed demultiplexing for raw Illumina reads at the IBEST Genomics Core. We used only forward reads (i.e., ITS1) in our analyses because the forward reads had higher quality than the reverse reads based on the following steps[10]. We used FastQC[15] to visualize read quality and the -fastq_eestats2 command in USEARCH v. 10[16] to calculate the number of reads that would pass given filters based on expected error rates and length thresholds. We filtered and trimmed the reads at a max expected error of 0.5 and length of 200 bp[11]. We dereplicated the reads with the command - fastx_uniques (parameters - sizeout) and removed singletons and rare reads via the commands -unoise3 (parameters –zotus - minsize 8) in USEARCH v.10[10,11,16]. Overall we obtained 4,945,487 reads prior to quality control, and 1,529,284 reads after quality control.

Taxonomy assignment

We performed taxonomic assignment for each OTU with the SINTAX algorithm[17] based on the UNITE database (version 7.2[18], with a cut-off of 0.8 for class-level assignments to be included in analyses of taxonomic abundance[19]). We used only OTUs representing Ascomycota for further analyses because foliar endophyte communities in tropical forests are dominated by Ascomycota as observed in our data (75% of reads and 62% of OTUs were assigned to Ascomycota; see also ref. [20]). The reads that were not assigned to Ascomycota were assigned to Basidiomycota (7.1%) and unidentified (17.9%). Taxonomic data are shown in Supplementary Data 3 and Supplementary Fig. 4.

Rarefaction

We subsampled reads from each sample until the coverage was 97.9% to reduce artifactual effects of read number on richness[21]. We confirmed similar results for representative analyses between coverage-based rarefaction and rarefaction to 10,000 reads per sample (approximating the read number from each sample, for which the median was 12,561 reads[22]) and rarefaction to the minimum read number of all samples (i.e., 1,000 reads per sample, Supplementary Fig. 1).

Statistical analyses

Prior to analyses we logarithm-transformed endophyte richness, total phenolics, flavonoids, and LMA. We logit-transformed the relative concentrations of condensed tannins after adjusting 0 and 1 values by 0.0001 to ensure finite values[23]. Due to multicollinearity among total phenolics, total flavonoids, and condensed tannins (VIFs > 2), we used PCA to define the first two PCs with these chemical contents and LMA (Supplementary Data 2, Supplementary Table 1). Chemical defense explained 57.3% of all variation. Physical defense explained 23.1% of all variation.

Details of the factors used in variation partitioning

We included a spatial factor in variation partitioning to account for factors that were not measured here but are structured spatially[24]. To compute the spatial factor, we computed principal coordinates of neighbor matrices based on the geographic distances among sites with pcnm function in the R package *vegan*[25] version 2.5-2 and extracted three spatial eigenvectors with forward selection ($P < 0.05$).

We used PCA to summarize vegetation to avoid multicollinearity. Five vegetation factors that explained variation observed on plots were chosen via PCA: host phylogenetic diversity, stem richness, canopy cover, canopy height, and basal area. To define phylogenetic diversity of hosts we used *picante*[26] to calculate the phylogenetic species variability metric (PSV) based on plants collected in each plot[27,28]. We used the first two PCs, which explained 42.9% and 32.7% of the total variation in vegetation, to represent a vegetation factor in the analysis. Loadings of each PC for climate and vegetation factors are shown in Supplementary Table 2.

To account for traits structured by host phylogeny but not evaluated explicitly in our measurements (e.g., leaf carbon content, leaf nitrogen concentration, or other factors[29,30]), we included host phylogeny as host phylogeny eigenvectors (HPEs) in our model. We extracted the phylogenetic tree for host plants from PHYLOMATIC v.3 (phylodiversity.net/phylomatic[31] and used the PVRdecomp function in *PVR* package of R to compute HPEs[32–34]. Five HPEs were selected by the correlation with endophyte species richness and forward selection ($P < 0.05$) with the *packfor* package in R[32,33,35]. We combined the selected HPEs and 2 PCs from chemical and physical defense to define the host factor for analyses. All statistical analyses for which methods were not mentioned above (e.g., PCA) were performed in JMP (versions 12 and 13, SAS Institute, Cary, NC, USA).

Supplementary Figures

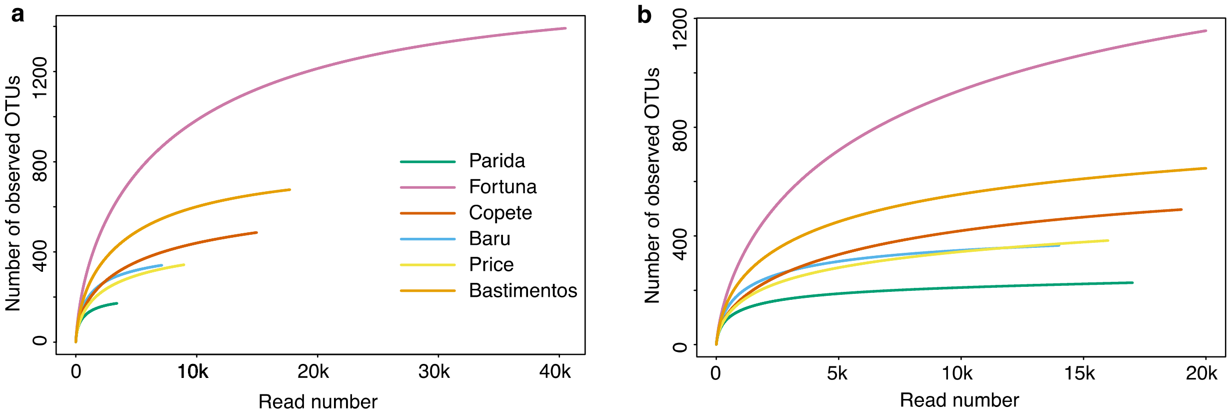

Supplementary Fig. 1. Rarefaction curves of the number of observed OTU in each site (**a**) after coverage-based rarefaction, and (**b**) rarefaction into 1,000 reads per sample (i.e., the minimum read number among all samples). Each curve is colored by site. Both approaches showed the same pattern of species richness, and demonstrated that our sampling was sufficient to support the analyses outlined in the manuscript (n = 106 biologically independent samples).

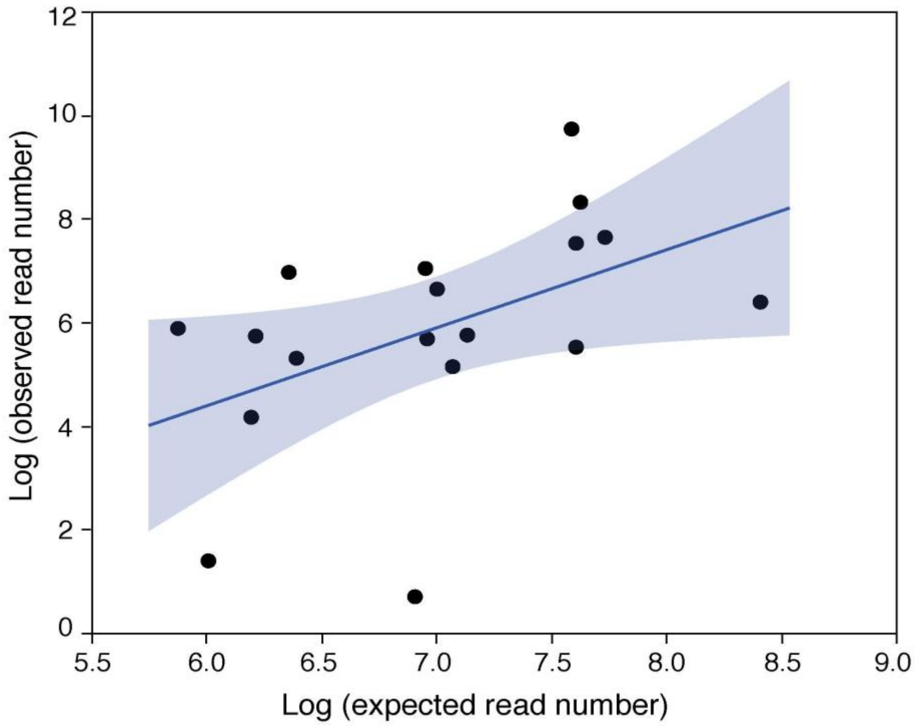

Supplementary Fig. 2. Expected and observed read number from Ascomycota in the tiered mock community were associated positively ($R^2 = 0.23$, $P = 0.04$), leading us to use both presence/absence data and relative abundance data for calculation of Jaccard and Morisita indices, respectively. Data were log-transformed for normality (Shapiro-Wilk W test for goodness-of-fit, $P > 0.1$ in both cases). Shaded area indicates 95% confidence interval.

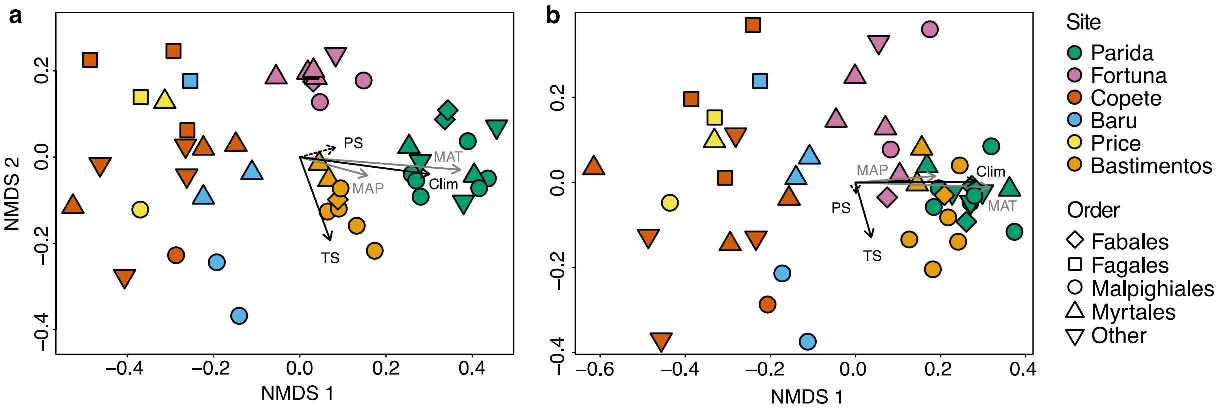

Supplementary Fig. 3. Endophyte community composition as a function of climate and seasonality when evaluated on the basis of (**a**) presence-absence data (Jaccard index) and (**b**) relative abundance data (Morisita index) for Rosanae (n = 48 biologically independent samples). Each dot represents an endophyte community from an individual plant. Colors correspond to sites and shapes correspond to orders of host plants. Black arrows represent the significant vectors of climate PC1 (Clim, considering MAT and MAP) and temperature seasonality (TS) fitted to the ordination scale (**a**: $P < 0.001$ and $R^2 = 0.84$, $P = 0.002$ and $R^2 = 0.25$, respectively; **b**: $P < 0.001$ and $R^2 = 0.86$, $P = 0.03$ and $R^2 = 0.16$, respectively). Grey arrow represents the vector of each climate factor (MAT and MAP) (**a**: $R^2 = 0.83$ and 0.30; **b**: $R^2 = 0.84$ and 0.41, respectively. $P < 0.001$ in each case), and the dotted arrow represents the vector of precipitation seasonality ($P > 0.1$ in each case).

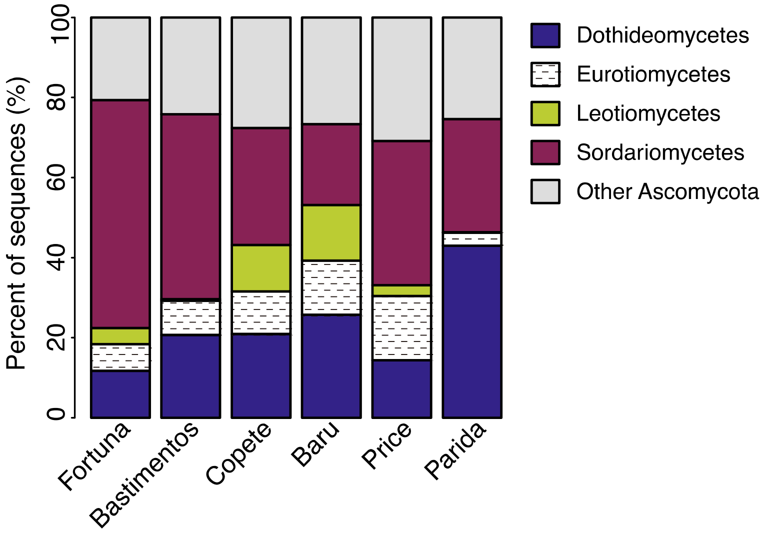

Supplementary Fig. 4. Abundance of the most commonly observed classes of Ascomycota among endophytes at each site. Each bar was ordered by temperature seasonality, in ascending order (n = 106 biologically independent samples).

Supplementary Tables

Supplementary Table 1. Loadings of each factor on the first two principal components representing chemical and physical defenses, from principal component analysis with total phenolics, flavonoids, condensed tannin and leaf mass per area (LMA). Phenolics, flavonoids, and tannins were treated as chemical defenses, and LMA as a physical defense.

**Defense**	**PC1 (chemical defense)**	**PC2 (physical defense)**
**Phenols**	0.87	-0.12
**Flavonoids**	0.87	-0.12
**Condensed tannins**	0.81	-0.15
**LMA**	0.36	0.93

Supplementary Table 2. Loadings of each factor on the first two principal components representing (A) climate and (B) vegetation by PCA.

(A)	PC1	PC2
MAT	0.93549	0.35335
MAP	0.93549	-0.35335

(B)	PC1	PC2
Host phylogenetic diversity	0.46249	-0.52812
Stem richness	0.6705	0.59914
Canopy cover	0.54999	0.73978
Canopy height	-0.53215	0.62633
Basal area	-0.94520	0.24444

Supplementary Table 3. Effects of each explanatory variable on endophyte community composition and structure, for analyses based on Jaccard and Morisita index, respectively.

Term	Community composition (Jaccard index)			Community structure (Morisita index)		
		Variation explained (%)			Variation explained (%)	
	P-value	By itself	with other factors	P-value	By itself	with other factors
**Seasonality**	0.001	1.8	6.2	0.001	1.8	4.6
**Spatial factor**	0.001	1.3	5.6	0.001	1.3	4.9
**Climate**	0.001	0.8	4.1	0.001	0.9	5.7
**Vegetation**	0.001	0.9	5.1	0.002	0.9	4.1
**Host factor**	0.011	0.4	9.6	0.002	1.7	5.7
**Total variation explained by all factors**			13.1			14.0

Supplementary Table 4. Number of unique and shared OTUs at each site.

Site	# of unique OTUs	# of OTUs shared with another site	Species richness	% of unique OTU
**Parida**	99	73	172	57.6
**Fortuna**	1123	269	1392	80.7
**Copete**	256	230	486	52.7
**Baru**	180	161	341	52.8
**Price**	177	166	343	51.6
**Bastimentos**	435	241	676	64.3

Supplementary References

1. Pérez-Harguindeguy, N. *et al.* New handbook for standardised measurement of plant functional traits worldwide. *Aust. J. Bot.* **61**, 167–234 (2013).
2. Kurokawa, H., Peltzer, D. A. & Wardle, D. A. Plant traits, leaf palatability and litter decomposability for co-occurring woody species differing in invasion status and nitrogen fixation ability. *Funct. Ecol.* **24**, 513–523 (2010).
3. Ainsworth, E. A. & Gillespie, K. M. Estimation of total phenolic content and other oxidation substrates in plant tissues using Folin–Ciocalteu reagent. *Nat. Protoc.* **2**, 875–877 (2007).
4. Heimler, D., Vignolini, P., Dini, M. G. & Romani, A. Rapid tests to assess the antioxidant activity of *Phaseolus vulgaris* L. dry beans. *J. Agric. Food Chem.* **53**, 3053–3056 (2005).
5. Porter, L. J., Hrstich, L. N. & Chan, B. G. The conversion of procyanidins and prodelphinidins to cyanidin and delphinidin. *Phytochemistry* **25**, 223–230 (1985).
6. Schofield, P., Mbugua, D. M. & Pell, A. N. Analysis of condensed tannins: a review. *Anim. Feed Sci. Technol.* **91**, 21–40 (2001).
7. Waterman, P. G. & Mole, S. *Analysis of phenolic plant metabolites*. vol. 83 (Blackwell Scientific Oxford, 1994).
8. U'Ren, J. M. & Arnold, A. E. 96 well DNA extraction protocol for plant and lichen tissue stored in CTAB. *protocols.io* (2017) doi:10.17504/protocols.io.fscbnaw.
9. Singer, V. L., Jones, L. J., Yue, S. T. & Haugland, R. P. Characterization of PicoGreen reagent and development of a fluorescence-based solution assay for double-stranded DNA quantitation. *Anal. Biochem.* **249**, 228–238 (1997).

10. Sarmiento, C. *et al.* Soilborne fungi have host affinity and host-specific effects on seed germination and survival in a lowland tropical forest. *Proc. Natl. Acad. Sci. U. S. A.* **114**, 11458–11463 (2017).

11. Daru, B. H., Bowman, E. A., Pfister, D. H. & Arnold, A. E. A novel proof of concept for capturing the diversity of endophytic fungi preserved in herbarium specimens. *Philos. Trans. R. Soc. Lond. B Biol. Sci.* **374**, (2018).

12. Naranjo-Ortiz, M. A. & Gabaldón, T. Fungal evolution: diversity, taxonomy and phylogeny of the fungi. *Biol Rev* **94**, 2101–2137 (2019).

13. Nguyen, N. H., Smith, D., Peay, K. & Kennedy, P. Parsing ecological signal from noise in next generation amplicon sequencing. *New Phytol.* **205**, 1389–1393 (2015).

14. Huang, Y.-L. *et al.* Using collections data to infer biogeographic, environmental, and host structure in communities of endophytic fungi. *Mycologia* **110**, 47–62 (2018).

15. Andrew, S. FastQC: a quality control tool for high throughput sequence data. (2010).

16. Edgar, R. C. Search and clustering orders of magnitude faster than BLAST. *Bioinformatics* **26**, 2460–2461 (2010).

17. Edgar, R. C. SINTAX: a simple non-Bayesian taxonomy classifier for 16S and ITS sequences. *bioRxiv* 074161 (2016).

18. Kõljalg, U. *et al.* Towards a unified paradigm for sequence-based identification of fungi. *Mol. Ecol.* **22**, 5271–5277 (2013).

19. Edgar, R. C. Accuracy of taxonomy prediction for 16S rRNA and fungal ITS sequences. *PeerJ* **6**, e4652 (2018).

20. Arnold, A. E. & Lutzoni, F. Diversity and host range of foliar fungal endophytes : are tropical leaves biodiversity hotspots? *Ecology* **88**, 541–549 (2007).

21. Chao, A. & Jost, L. Coverage-based rarefaction and extrapolation: standardizing samples by completeness rather than size. *Ecology* **93**, 2533–2547 (2012).

22. de Cárcer, D. A., Denman, S. E., McSweeney, C. & Morrison, M. Evaluation of subsampling-based normalization strategies for tagged high-throughput sequencing data sets from gut microbiomes. *Appl. Environ. Microbiol.* **77**, 8795–8798 (2011).

23. Moles, A. T. *et al.* Correlations between physical and chemical defences in plants: tradeoffs, syndromes, or just many different ways to skin a herbivorous cat? *New Phytol.* **198**, 252–263 (2013).

24. Borcard, D. & Legendre, P. Environmental control and spatial structure in ecological communities: an example using oribatid mites (Acari, Oribatei). *Environ. Ecol. Stat.* **1**, 37–61 (1994).

25. Oksanen, J. *et al.* Vegan: community ecology package, version 2.5-2. (2018).

26. Webb, C. O., Ackerly, D. D. & Kembel, S. W. Phylocom: software for the analysis of phylogenetic community structure and trait evolution. *Bioinformatics* **24**, 2098–2100 (2008).

27. Pearse, W. D., Purvis, A., Cavender-Bares, J. & Helmus, M. R. Metrics and models of community phylogenetics. in *Modern Phylogenetic Comparative Methods and Their Application in Evolutionary Biology: Concepts and Practice* (ed. Garamszegi, L. Z.) 451–464 (Springer Berlin Heidelberg, 2014).

28. Nguyen, N. H. *et al.* Ectomycorrhizal fungal diversity and saprotrophic fungal diversity are linked to different tree community attributes in a field-based tree experiment. *Mol. Ecol.* **25**, 4032–4046 (2016).

29. Kraft, N. J. B. & Ackerly, D. D. Functional trait and phylogenetic tests of community assembly across spatial scales in an Amazonian forest. *Ecol. Monogr.* **80**, 401–422 (2010).

30. Baraloto, C. *et al.* Using functional traits and phylogenetic trees to examine the assembly of tropical tree communities. *J. Ecol.* **100**, 690–701 (2012).

31. Zanne, A. E. *et al.* Corrigendum: Three keys to the radiation of angiosperms into freezing environments. *Nature* **521**, 380 (2015).

32. Diniz-Filho, J. A. F. *et al.* On the selection of phylogenetic eigenvectors for ecological analyses. *Ecography* **35**, 239–249 (2012).

33. Burns, J. H., Anacker, B. L., Strauss, S. Y. & Burke, D. J. Soil microbial community variation correlates most strongly with plant species identity, followed by soil chemistry, spatial location and plant genus. *AoB Plants* **7**, (2015).

34. Shao, J. *et al.* Plant evolutionary history mainly explains the variance in biomass responses to climate warming at a global scale. *New Phytol.* **222**, 1338–1351 (2019).

35. Dray, S., Legendre, P. & Blanchet, G. packfor: Forward Selection with permutation (Canoco p. 46). *R package version 0. 0-7/r58* (2009).
