# Supplementary material for: Climate and seasonality drive the richness and composition of tropical fungal endophytes at a landscape scale
Source: Commun Biol. 2021 Mar 9;4:313. doi: 10.1038/s42003-021-01826-7 (PMC7943826; doi:10.1038/s42003-021-01826-7)
Supplement: Supplementary file 2 — Description of Additional Supplementary Files [file 42003_2021_1826_MOESM2_ESM.pdf]

Description of Additional Supplementary Files

File Name: Supplementary Data 1

Description: Vegetation characteristics of each plot surveyed for endophytic fungi in western Panama. Stem density is the total number of living stems with > 1cm diameter at breast height (DBH). Stem richness was categorical and based on morphology: 1, <2 species; 2, 2-3 species; 3, 4-5 species; and 4, > 5 species greater than 1 cm DBH on the 4 x 5 m plot. Canopy cover was measured with a densiometer at nine points on each plot (four corners and four middle points on each edge, and the center of the plot). Aspect was evaluated with a compass toward the direction that a slope faced. It varied from 0 (no rise) through 180 (the slope facing south) to 360 (north).

File Name: Supplementary Data 2

Description: Plant species collected at each site, with measures of DNA concentration, plant characteristics (height, diameter at breast height (DBH)), distance to the nearest conspecific, leaf mass per area (LMA), total phenolics, flavonoids, condensed tannins, measures of DNA concentration, and endophyte richness. Plant classification followed the TROPICOS system based on the Angiosperm Phylogeny Group (APG III) classification updated by the APG in 2016 (APG IV). Voucher specimens of all plants have been deposited at the Herbarium of the University of Panama. This list includes only the host species used in the final analyses. We determined that within two focal sites, representative angiosperms did not differ meaningfully from one another in leaf chemical or physical defense (Parida, chemical defenses: $F_{1,11} = 0.8125$, $P = 0.3867$; physical defense: $F_{1,11} = 1.8817$, $P = 0.1975$); Bastimentos, chemical defenses: $F_{1,6} = 0.7104$, $P = 0.4316$; physical defense: $F_{1,6} = 0.7135$, $P = 0.4307$). We compared leaf defenses between Parida and Bastimentos via multiple regression analyses with PC1 (chemical defense) or PC2 (physical defense) as a function of site, with host species treated as a random effect. Analyses were conducted at the site level, with data pooled for the two plots in each site. To evaluate differences in endophyte richness between Parida and Bastimentos, we first took variation due to PC1 into account, as representative leaves in each site differed in their degree of chemical defense. From a regression of PC1 and log species richness of endophytes we retained residuals, which were then used as the dependent variable for a regression analysis with species as a random effect and site as a fixed effect.

File Name: Supplementary Data 3

Description: Assigned class and genus of each endophyte OTU with the SINTAX algorithm based on the UNITE database (version 7.2) with an inferred confidence level. Operational taxonomic units (OTUs) were assigned at 95% sequence similarity of ITS1 region. We used a cutoff of 0.8 for class identification for the taxonomy abundance analysis. The assigned class or genus with lower than 0.8 in a confidence level (shown here as N/A) was included as "Other Ascomycota". We did not use shallow taxonomic levels (i.e., species) in our analyses due to the short length of MiSeq sequences.

File Name: Supplementary Data 4
Description: Ascomycota included in the mock communities (tiered, even), with DNA concentration and expected and observed read numbers.
