# Supplementary material for: Climate and seasonality drive the richness and composition of tropical fungal endophytes at a landscape scale
Source: Commun Biol. 2021 Mar 9;4:313. doi: 10.1038/s42003-021-01826-7 (PMC7943826; doi:10.1038/s42003-021-01826-7)
Supplement: Supplementary file 7 — Reporting Summary [file 42003_2021_1826_MOESM7_ESM.pdf]

nature research

Corresponding author(s): A. Elizabeth Arnold

Last updated by author(s): Jan 7, 2021

Reporting Summary

Nature Research wishes to improve the reproducibility of the work that we publish. This form provides structure for consistency and transparency in reporting. For further information on Nature Research policies, see our Editorial Policies and the Editorial Policy Checklist.

Statistics

For all statistical analyses, confirm that the following items are present in the figure legend, table legend, main text, or Methods section.

n/a	Confirmed	
☐	☒	The exact sample size (n) for each experimental group/condition, given as a discrete number and unit of measurement
☐	☒	A statement on whether measurements were taken from distinct samples or whether the same sample was measured repeatedly
☐	☒	The statistical test(s) used AND whether they are one- or two-sided<br>*Only common tests should be described solely by name; describe more complex techniques in the Methods section.*
☐	☒	A description of all covariates tested
☐	☒	A description of any assumptions or corrections, such as tests of normality and adjustment for multiple comparisons
☐	☒	A full description of the statistical parameters including central tendency (e.g. means) or other basic estimates (e.g. regression coefficient) AND variation (e.g. standard deviation) or associated estimates of uncertainty (e.g. confidence intervals)
☐	☒	For null hypothesis testing, the test statistic (e.g. F, t, r) with confidence intervals, effect sizes, degrees of freedom and P value noted<br>*Give P values as exact values whenever suitable.*
☒	☐	For Bayesian analysis, information on the choice of priors and Markov chain Monte Carlo settings
☒	☐	For hierarchical and complex designs, identification of the appropriate level for tests and full reporting of outcomes
☒	☐	Estimates of effect sizes (e.g. Cohen's d, Pearson's r), indicating how they were calculated

Our web collection on statistics for biologists contains articles on many of the points above.

Software and code

Policy information about availability of computer code

Data collection	We used standard, publicly available software for Illumina quality control, taxonomic identification, etc. All are stated clearly in the manuscript.
Data analysis	We used standard packages in R and the standard version of JMP for statistical analyses.

For manuscripts utilizing custom algorithms or software that are central to the research but not yet described in published literature, software must be made available to editors and reviewers. We strongly encourage code deposition in a community repository (e.g. GitHub). See the Nature Research guidelines for submitting code & software for further information.

Data

Policy information about availability of data

All manuscripts must include a data availability statement. This statement should provide the following information, where applicable:

- Accession codes, unique identifiers, or web links for publicly available datasets
- A list of figures that have associated raw data
- A description of any restrictions on data availability

Sequences used in this study are deposited in NCBI Sequence Read Archive (SRA) under BioSample accession ID SAMN15675876-SAMN15675983 of SRA accession PRJNA649645. All metadata, other data types and codes used in this study are publicly available in figshare (see main manuscript).

Field-specific reporting

Please select the one below that is the best fit for your research. If you are not sure, read the appropriate sections before making your selection.

☐ Life sciences ☐ Behavioural & social sciences ☒ Ecological, evolutionary & environmental sciences

For a reference copy of the document with all sections, see nature.com/documents/nr-reporting-summary-flat.pdf

Ecological, evolutionary & environmental sciences study design

All studies must disclose on these points even when the disclosure is negative.

Study description	We measured environmental characteristics of 12 study plots, leaf chemistry (phenolics, tannins, flavonoids) in representative angiosperms from each plot, gathered climate data from online databases, and surveyed endophytes in leaves.
Research sample	Each research sample is a leaf sample from a tropical tree on one of 12 study plots. The number of trees and species is presented in the manuscript and detailed extensively in the supplementary tables. The leaves were sampled contemporaneously in all sites.
Sampling strategy	Leaf samples were collected fresh and processed rapidly to preserve material for DNA extraction and chemical analysis. The sample sizes were based on contemporaneous work in a lowland forest in Panama (Tellez et al. in review) and previous work in Panama and in subtropical, temperate, and boreal forests (U'Ren et al. 2019, 2016, 2014, 2012; Arnold & Lutzoni 2007; Arnold et al. 2009), taking into account species accumulation curves and estimated species richness.
Data collection	Lead author Oita collected leaves with Arnold and other authors in the field, measured environmental characteristics, and processed leaves for DNA extraction and Illumina sequencing. Oita gathered all data on leaf chemistry and obtained climate data online. Plants were identified by Ibanez. Arnold and Oita led data analyses.
Timing and spatial scale	Field samples were collected contemporaneously within a period of a few weeks in June/July 2016. The spatial scale is described in full in the manuscript; our plots ranged from sea level to ca. 3000 m, and from the Pacific Ocean to the Caribbean Sea.
Data exclusions	No data were excluded except for those specifically relevant to quality control of Illumina sequences (identified as problematic based on our strict positive and negative controls in the sequencing process) or obvious outliers ≥ 2 SD from the mean in quantitative analyses.
Reproducibility	All protocols are published or are described in full here. Our public release of the positive control (mock community) provides a calibration tool. All analysis methods are public/with standard software. We retained vouchers of all plant specimens.
Randomization	NA
Blinding	All data were collected blindly with regard to plant species, climate data, and leaf chemistry.

Did the study involve field work? ☒ Yes ☐ No

Field work, collection and transport

Field conditions	Field sites ranged from lowland tropical forests on the Pacific and Caribbean coasts to highland forests at Volcan Baru, Panama. Climate data are provided in detail in the paper, as climate is central to our study framework.
Location	Panama, in the provinces of Chiriqui and Bocas del Toro.
Access & import/export	All collections were made with express permission from the Ministry of the Environment of Panama. All imports were made with express permission of the USDA APHIS PPQ.
Disturbance	Our collections, in remote areas typically not visited by others, involved minimal visible or physical damage to trees.

Reporting for specific materials, systems and methods

We require information from authors about some types of materials, experimental systems and methods used in many studies. Here, indicate whether each material, system or method listed is relevant to your study. If you are not sure if a list item applies to your research, read the appropriate section before selecting a response.

Materials & experimental systems

n/a	Involved in the study
☒	☐ Antibodies
☒	☐ Eukaryotic cell lines
☒	☐ Palaeontology and archaeology
☒	☐ Animals and other organisms
☒	☐ Human research participants
☒	☐ Clinical data
☒	☐ Dual use research of concern

Methods

n/a	Involved in the study
☒	☐ ChIP-seq
☒	☐ Flow cytometry
☒	☐ MRI-based neuroimaging
